# Education and training for medicines development, regulation, and clinical research in emerging countries

**DOI:** 10.3389/fphar.2015.00080

**Published:** 2015-04-14

**Authors:** Sandor Kerpel-Fronius, Bernd Rosenkranz, Elizabeth Allen, Rolf Bass, Jacques D. Mainard, Alex Dodoo, Dominique J. Dubois, Mandisa Hela, Steven Kern, Joao Massud, Honorio Silva, Jeremy Whitty

**Affiliations:** ^1^Department of Pharmacology and Pharmacotherapy, Faculty of Medicine, Semmelweis University, Budapest, Hungary; ^2^Division of Clinical Pharmacology, Faculty of Medicine and Health Sciences, Stellenbosch University, Cape Town, South Africa; ^3^Fundisa African Academy of Medicines Development, Cape Town, South Africa; ^4^Division of Clinical Pharmacology, Department of Medicine, South African Faculty, Global Health Network, University of Cape Town, Cape Town, South Africa; ^5^University of Basel, Pharmaceutical Medicine, Basel, Switzerland; ^6^Ministry of Higher Education and Research, Institut National de la Santé et de la Recherche Médicale, Paris, France; ^7^WHO Collaborating Centre for Advocacy and Training in Pharmacovigilance, Centre for Tropical Clinical Pharmacology and Therapeutics, University of Ghana Medical School, Accra, Ghana; ^8^Postgraduate Programme in Pharmaceutical Medicine and Medicines Development Sciences, Université Libre de Bruxelles, Brussels, Belgium; ^9^Medicines Control Council, Pretoria, South Africa; ^10^Quantitative Sciences, Bill & Melinda Gates Foundation, Seattle, WA, USA; ^11^Education and Research Institute, Hospital Sirio-Libanes, Sao Paulo, Brazil; ^12^BioPharma Educational Initiative, Rutgers University School of Health Related Professions, Newark, NJ, USA; ^13^Inter American Foundation for Clinical Research, New York, NY, USA; ^14^School of Health Sciences, Hibernia College, Dublin, Ireland

**Keywords:** pharmaceutical medicine, clinical trials, regulatory agencies, low and middle income countries, competencies, learning outcomes

## Abstract

The aim of this satellite workshop held at the 17th World Congress of Basic and Clinical Pharmacology (WCP2014) was to discuss the needs, optimal methods and practical approaches for extending education and teaching of medicines development, regulation, and clinical research to Low and Middle Income Countries (LMICs). It was generally agreed that, for efficiently treating the rapidly growing number of patients suffering from non-communicable diseases, modern drug therapy has to become available more widely and with a shorter time lag in these countries. To achieve this goal many additional experts working in medicines development, regulation, and clinical research have to be trained in parallel. The competence-oriented educational programs designed within the framework of the European Innovative Medicine Initiative-PharmaTrain (IMI-PhT) project were developed with the purpose to cover these interconnected fields. In addition, the programs can be easily adapted to the various local needs, primarily due to their modular architecture and well defined learning outcomes. Furthermore, the program is accompanied by stringent quality assurance standards which are essential for providing internationally accepted certificates. Effective cooperation between international and local experts and organizations, the involvement of the industry, health care centers and governments is essential for successful education. The initiative should also support the development of professional networks able to manage complex health care strategies. In addition it should help establish cooperation between neighboring countries for jointly managing clinical trials, as well as complex regulatory and ethical issues.

## Introduction

Global educational efforts appropriately focused according to local needs are required for supporting science-based rational therapy with conventional and biological agents, vaccines, and drug-medical device combinations worldwide. The 17th World Congress of Basic and Clinical Pharmacology in Cape Town, South Africa offered a unique opportunity to discuss with experts working in differently developed environments the current state and challenges of education and training for medicine development, regulation, and clinical research in Low and Middle Income Countries (LMICs). The goals were to understand and appraise the magnitude of the educational needs of lesser developed countries and to formulate teaching concepts for covering the identified topics in the respective scientific fields. Finally, the practical approach and financial backing needed to realize such programs were discussed to appraise possible risks involved. The satellite workshop was organized in cooperation with the Fundisa African Academy of Medicines Development and the Hungarian Society for Experimental and Clinical Pharmacology, under the joint scientific auspices of the Innovative Medicine Initiative-PharmaTrain project [IMI-PhT] (2015) and the International Federation of Associations of Pharmaceutical Physicians and Pharmaceutical Medicine [IFAPP] (2015). The lecturers were selected to represent regional experts working at local universities, regulatory agencies, pharmaceutical companies and other organizations. Other speakers were recruited from PhT course providers and IFAPP member organizations engaged in educational activities in various regions of the world. The topics of the workshop were divided into five subject areas which will be discussed separately below.

## Workshop Topics

### Educational Needs of a Changing Health Care Environment in LMIC’s

Over the past 5 years the educational programs of the various medicine development courses offered in Europe were updated and harmonized within the IMI-PhT project. For this education program the entire medicines development process from molecule to marketplace and health care application was captured in a syllabus containing 180 topics (PhT Syllabus; PharmaTrain, 2015b). The topics were organized into six basic modules which can be presented in 3–4 days at suitable intervals such that students may attend the course alongside full time employment. Sixty learning outcomes were specified which have to be mastered by the students to fulfill the competency requirements for becoming an expert in medicine development. The learning outcomes are considered to be the backbone for harmonizing the education program of the different course providers. Many similarly structured elective modules were also produced to fulfill additional educational requirements for further specialization. Finally, a set of tools for the implementation and assessment of shared quality standards were developed. In cooperation with other organizations, two further programs having essentially similar education principles were developed for teaching drug regulatory science and clinical investigation within the PhT cooperation (PhT Manual; PharmaTrain, 2015a).

The IMI-PhT program was primarily developed for countries having well developed, large innovative pharmaceutical industry where many specialists of medicines development and regulation require high level education for efficiently participating in the very competitive industrial working environment. It was obvious that this elaborate program cannot be transferred in its original form to LMICs which have lesser developed pharmaceutical industries, regulatory agencies and a smaller number of trained clinical investigators. To provide useful training for these communities it is a necessary first step to adapt the PhT educational concept and materials to the individual needs of the targeted countries. Furthermore, in preliminary consultations it was unanimously agreed that medicines development, regulatory science and clinical investigation must be taught in conjunction with each other; high level local medical research and efficient drug therapy can be promoted only if these three equally important and mutually supportive pillars are built in parallel. The meeting focused on postgraduate education designed for experts who have previously finished higher level training. However, it was acknowledged that, especially in some low income countries, local workers with lesser qualification must also be trained to initiate local activities.

In the introductory lecture Kerpel-Fronius explained the rational for concentrating the discussion on issues primarily related to medicines for non-communicable diseases (NCD) like cardio-vascular diseases, diabetes, cancer, etc. This decision was based on the rapidly changing composition of the local population which leads to stratified needs for medical care (Table 1). The communicable diseases require specific scientific attention, including the discovery and development of new medicines, large scale epidemiologic interventions, primary preventions, etc. Generally these topics are addressed by disease oriented projects sponsored by various organizations. Their educational programs have to be evaluated and tailored individually according to the nature of the disease. In parallel with improving economies a rapidly emerging urban population with increased life expectancy has been developing in LMICs in recent years. They live in an improved environment, suffer increasingly from NCD and have expanding access to higher quality, more sophisticated medical care using modern equipment and innovative medicines. Nevertheless, according to the data of the WHO, 85% of premature deaths from NCDs occur in developing countries (WHO, 2013b). The availability of modern medicines must therefore be improved, their regulatory approval must be accelerated and finally their proper use by medical staff must be evaluated and practiced within the local health care infrastructure. Special attention must be given to the major safety and regulatory problems presented by the new biological medicinal agents and their follow-on biosimilar preparations produced in many countries in very different qualities. Hospitals and regulatory agencies need additional well trained experts to handle these agents properly.

**TABLE 1:** **Different medical needs of the various populations present in LMIC’s**.

Higher income population	Low income population
Well educated	Poorly educated
Can afford modern medical care	May live in an unhygienic environment and cannot afford modern medical care
Access to health-care is similar or occasionally even better than in developed countries. Demand and require modern, well equipped medical facilities and highly effective medicines to treat broad range of illnesses. Medical personnel who can apply up-to-date treatment, can participate in international trials and can organize local clinical trials	Access to health-care is limited Receives generally poorer medical care since the delivery of modern therapeutic methods is made difficult due to large distances and a lack of infrastructure. Suffers primarily from communicable diseases. Special organization and specially trained personnel is needed to treat NCD affecting a large population
Regulatory Agency which can adequately handle both modern sophisticated medicines and an increased work load caused by innovative and many follow-on chemical and biological medicines	

According to Dodoo from Ghana the environment is ripe for the development and deployment of new health technologies in LMICs. In general, commitments are plenty; however, few countries have either qualitatively or quantitatively adequate capacity for drug/vaccine testing and regulation. Although partnerships are urgently required to perform these tasks, the emerging economies cannot leave the development of viable and locally-relevant solutions to external partners only. More specialists in drug development and regulation are urgently needed in Africa. To reach this goal, besides specialists in molecular biology, immunology, and pharmacology, statisticians, pharmaco- and health care economists, lawyers, and ethicists have to be trained to deal with both the scientific and related social, legal issues of medical care. All stakeholders in such training must ensure that the programs are relevant, useful, cost-effective and modular to enable training alongside working. Finally the courses should provide internationally acceptable certificates. According to Dodoo, training institutions in emerging economies have to get involved in practical day-to-day activities of the respective agencies, companies and departments dealing with drug discovery, research and regulation. Effective cooperation between the industry, healthcare centers and government is essential to reach these goals. Efforts should be made to ensure that any approach utilized is sustainable and cost-effective. Continent-wide initiatives, when properly harnessed, have the highest chance of success and sustainability.

Dealing with specific healthcare problems and related educational activities in LMICs is an area in which the Bill & Melinda Gates Foundation has a long and outstanding track record. For many years it has supported global health product development networks built on partnership, expertise, promoting improved performance and the right products for the right patients at the right time. The primary aim of the Integrated Development Group is to augment the capabilities of program strategy teams and partners with end-to-end product development. It promotes integrated medicines development combining quantitative sciences, regulatory affairs, diagnostics, chemistry, manufacturing and controls. Several educational collaborations with many outstanding scientific organizations have been organized to teach clinical pharmacology, clinical and regulatory sciences (Bill & Melinda Gates Foundation, 2015).

### Competencies Needed Locally. Availability of Educational Infrastructure and Activities for Providing Advanced Continuous Professional Training in Medicines Development

Current problems with professional training in medicine development were addressed by Silva. He stated that there is a perceived mismatch between the profile of graduates from academic programs in the health care professions and the changing needs of the various health systems around the world. Professional education has not kept pace with these changes, largely because of fragmented, outdated and static curricula that produce ill-equipped graduates. Redesign of professional health education is thus necessary and timely. Usually, needs-oriented competence training is done within the framework of post-graduate and interprofessional education, where organizers of the various courses have much more freedom to combine different scientific disciplines and practical professional experience. The final goal should be to achieve the required competencies defined as “an observable ability of any professional, integrating multiple components such as knowledge, skills, values, and attitudes” (Frank et al., 2010). The main advantage of such competence based education is that the competencies of a given profession can be assessed to ensure their acquisition. Unfortunately the number of such courses in medicine development is low world-wide, and their scientific value and quality is frequently less well controlled compared to graduate education.

An IFAPP-Pharmatrain organized working group identified seven core competency domains for pharmaceutical medicine: discovery and early drug development; clinical development and clinical trials; medicines regulation; drug safety surveillance; ethics and subject protection; health care market place and finally communication and management skills. A total of 60 core competencies for Pharmaceutical Physicians and Drug Development Scientists were included within such domains and a Statement of Competence was prepared. Finally, the Learning Outcomes of the PharmaTrain Base Course were successfully aligned with the above mentioned competencies (Silva et al., 2013). Many other groups active in various fields of clinical research also defined their relevant competencies. These were recently aligned and harmonized by a Joint Task Force into a Harmonized Core Competency Framework for Clinical Research Professionals (Sonstein et al., 2014). It was concluded by the participants that the number of courses offered as part of the continuous professional development programs have to be increased and thoroughly planned to provide the necessary competencies aligned with the specific needs of LMICs. Further the needs may change over time. Therefore, a single approach for education and training is not feasible. The educational needs of the targeted populations should always be considered.

The advantages of e-learning were repeatedly emphasized since this method provides the broadest and most rapid access to teaching material worldwide. According to Whitty the availability of the internet seems not to be an issue in most places anymore. He pointed out that e-learning is especially suitable for postgraduate education since these trainees’ exhibit a special attitude to learning. Frequently they study besides working, come with different skills and life experience, are more autonomous adults, and their outcome expectations are different from those of graduate students. They are more goal oriented, so consequently the learning content must be relevant and practical enough to be used immediately in their jobs. According to his experience, blended learning, combining e-teaching material with tutorial support, is especially successful and leads to much higher completion rates compared to automated CPD.

Massud reported that in South America post-graduate training in pharmaceutical medicine is offered in Brazil, Argentina and Mexico. In the last two countries pharmaceutical medicine is accepted as a medical specialty. However, generally, medicines development and clinical pharmacology training at the universities are not considered as a priority. There is limited availability of postgraduate education for clinical investigators in the region. Most clinical research is carried out at the universities where the investigators are more science- than medicinal product-orientated. One post-graduate course in pharmaceutical medicine is offered in Brazil for which the initiation of an accreditation process by PharmaTrain is planned. A comparative evaluation of the training needs listed by people working in medicine development in Brazil and Peru showed interesting country differences in educational needs (Silva et al., 2014). More importantly, a very high need for training in business and management skills was registered in both countries. It is obvious that these subjects should be incorporated into the training programs of medicines development.

Rosenkranz discussed the important role played by South Africa in the development of new medicines on the African continent. Clinical trials (phase I–IV) have a long tradition in South Africa, and their number is rapidly increasing, with an estimated present value of about 1.3–2 billion Euro generated annually (Kahn and Gastrow, 2008). The typical patient populations available in first-world countries can also be accessed in South Africa. Due to the considerable burden of disease for HIV or TB, these patient populations are also available. Clinical trials are performed according to internationally accepted medical, ethical and regulatory standards. Clinical research in sub-Saharan Africa is mainly performed by international and local companies, but increasingly also by clinicians in the form of investigator-initiated trials in order to address gaps in knowledge about specific treatment requirements in the region. However, there is a clear need for more and better clinical research conducted in Africa, which should focus more evenly on the major contributors to burden of disease in this part of the world (Isaakidis et al., 2002; Siegfried et al., 2005).

Regulatory science and medicines development are not taught at undergraduate level in South Africa. However, several universities have developed bachelor and/or master courses, in regulatory science, drug development, pharmacoeconomics, pharmacovigilance, and pharmaceutical affairs. In 2010 a fully accredited 2 year diploma program in medicines development was started at the Division of Clinical Pharmacology, Stellenbosch University following the PhT concept, and a master’s program will be initiated in 2015. This program was accredited by PharmaTrain as a Center of Excellence in May 2014, as the first non-European training course. Non-academic training programs and workshops in medicines development and drug regulation are offered by the Fundisa African Academy of Medicines Development (FAAMD). Clinical Pharmacology has been recognized as a full medical specialty since 2009, comprising one of the Colleges of Medicine in South Africa (CMSA). In 2011, the UK Faculty of Pharmaceutical Medicine has exported its medical specialty examination, the Diploma in Pharmaceutical Medicine, to South Africa and Singapore. In South Africa, physicians working in the pharmaceutical industry are organized in the South African Association of Pharmaceutical Physicians [SAAPP] (under review) which had 83 members in 2005; this organization is currently being re-activated. The clinical research is organized in the South African Clinical Research Association (SACRA).

Unfortunately neither of these regionally-established courses attracts larger numbers of students from the neighboring countries, although this would be very helpful for promoting medicines research and regulation in smaller, lesser developed countries of the respective geographical regions. In Central-Eastern Europe this problem is being overcome by organizing an international-regional approach, primarily to offer easily accessible, relatively inexpensive international education for students working in several neighboring countries. Additionally, the course accepts students from all countries world-wide. The Cooperative European Medicine Development Course [CEMDC] (2015) organized with the support of the IMI-PhT program is an educational network in which 10 universities from 10 countries are cooperating. The educational program is jointly planned by the universities, and coordinated by the Semmelweis University in Hungary, which also issues the diplomas on behalf of the Kerpel-Fronius (2014) and Cooperative European Medicine Development Course [CEMDC] (2015). It was proposed that the organization of such regional networks could be both economically and scientifically very helpful also in LMIC’s for pulling together both the students and the local trainers as well as the scarce financial resources from a geographical area (Cooperative European Medicine Development Course [CEMDC], 2015).

### The Increased Qualitative and Quantitative Challenges of the Local Drug Regulatory Agencies

The increasing complexity of new therapeutic interventions, the new types of medicinal agents developed in recent years, presents a great challenge for competent authorities. Regulators can solve these problems only through increased collaboration between the various disciplines and extensive networking. Bass reported that, linked to the IMI-PhT project, a modular Master’s Program in Medicine Regulatory Affairs was organized jointly by the universities of Basel, Copenhagen and Kings College, London, in collaboration with several other organizations. The aim is to provide regulatory, industry and university professionals with a comprehensive understanding of the rapidly expanding regulatory requirements for medicines development and lifecycle management, along with competence to apply them. He suggested that similar cooperative regulatory educational programs would be needed also in emerging economies, since both the number of new, high tech medicinal products submitted for registration, as well as the local participation in international medicine development trials, will increase considerably in these regions. In addition they have to inspect drug manufacturing, and control international trade of medicinal products and active substances. An immediate solution might be to organize close cooperation between foreign competent authorities. However, the primary goal must be to train local experts by either sending them abroad to learn or, preferably, to establish local courses with international and national trainers.

In South Africa, The Medicines Control Council (MCC) is the statutory body that regulates new active substances, generic medicines and biologicals including vaccines, and oversees clinical trials as well as post-marketing variations. Hela explained that its present working model relies on external reviewers recruited mainly from universities and research institutions. This leads to long review timelines and little predictability when reviews will be completed. Over time, the workload has increased and become more complex, due to an increase in the number of regulatory submissions, particularly of generic medicines and post-marketing variation applications. In addition, the role of the regulator has expanded from its original focus on allopathic medicines to the inclusion of poorly regulated and unregulated commodities, e.g. complementary and alternative medicines, African Traditional Medicines, medical devices and *in vitro* diagnostics, food and cosmetics. As a consequence, it has been recognized that there is a need for a different regulatory model. A review of the MCC has been conducted with recommendations on strengthening internal capacity. Legislative amendments to this end are quite advanced and will lead to the formation of a new Agency, the South African Health Products Regulatory Agency (SAHPRA). The workload will be managed with a mixture of internally and externally performed reviews, with different proportions according to the various modules. A critical mass of capable reviewers is required to carry out the regulator’s mandate and obligations of ensuring timely access to safe, efficacious and quality commodities. This Agency therefore will require a new model of staff training, emphasizing capacity building through on the job training and *ad hoc* short courses. The need for training of regulatory experts in South Africa was assessed in a report prepared by the EU-funded Ecorys Health Consortium. They proposed establishment of a Regulatory Science Institute for coordinating training that should serve both the Agency and the industry in the region. A blended learning platform was recommended since on the job training alone is lengthy and takes much time from the established experts. The necessary training should be based on a gap analysis and be organized in cooperation with the universities and other training providers. Mentorship should also be introduced. Using this model the time for obtaining the necessary competence might be decreased from 18 to 24 months to about 6 months.

The recommendations for the South African regulatory Agency are relevant also to other African countries. Kern called the attention of the participants to the “innovation lag” that impacts uptake of new medicines, vaccines and medical innovations in LMIC’s. For a variety of reasons, these innovations face delays of varying and often unpredictable length before they are able to get to the people who can benefit from them. One contributing factor is the complex and un-harmonized regulatory process that exists for these innovations to gain entry into LMICs. Months and years can go by after innovations have already been approved by stringent regulatory authorities from upper income countries before the innovation receives prequalification from the WHO and local country regulatory approval. This impacts the ability of LMIC’s to both procure these innovations and distribute them to their citizens. The recently initiated WHO Collaborative Registration Procedure enables the exchange of confidential information about products, controlled according to the WHO Prequalification of Medicines Programme (PQP). The countries participating in the collaborative registration commit themselves for a national registration timeline of 90 days (WHO, 2013a). Related to this program the Bill & Melinda Gates Foundation (2015) facilitated with other partners and national governments the formation of six regional African regulatory harmonization blocks. As part of the program 44 harmonized guidelines and requirements were developed and underwent full approval at the ministerial level. It was proven that, through a joint registration procedure, the time to reach the market could be significantly decreased. It was extensively discussed that there are especially few experts who are able to deal with the new complex biological drugs in emerging countries (Matsoso et al., 2015). Not surprisingly the WHO promotes the formation of multinational organizations for making the regulatory evaluations jointly for countries in a given region (Rago, 2014).

### The Education of Clinical Investigators, Ethical Committees

The merits of an online professional community to provide affordable training around the globe for those working in clinical trials was convincingly demonstrated by Allen. Research in resource-limited settings has suggested that addressing technical competence, trial-specific training, knowledge-sharing, and experience-exchange for clinical investigators are key factors which would enable an increase in their confidence and motivation. The GlobalHealthTrials.org website, part of a broader Global Health Network, provides a forum to address these issues for clinical investigators and their teams. Sophisticated digital technology brings free, open access, peer-reviewed e-learning products and a professional membership scheme that supports career development in clinical trials. Since its start in May 2010 by Oxford University the website has become widely popular, with 230,000 visits recorded to date. It works well in practice since it addresses all regions, all staff roles and all disease areas. Furthermore it is democratic, neutral and does not belong to any one institution.

The concept and organization of the European Clinical Research Infrastructure Network (ECRIN) was demonstrated by Demotes-Mainard as a possible example to be copied in other geographical regions. The primary aim of this organization is to provide scientific and organizational background for performing non-industry funded clinical trials. These community sponsored trials are very important to address clinically relevant problems which are not directly related to marketing authorization of new medicines. Presently 23 countries are participating in the network. The elaborate organization provides information and consultancy during the preparation of trials and services during their conduct. A review carried out in the involved countries showed that for the clinical trials of new drugs the requirements for study initiation are very complex but quite similar. However, for medical devices, clinical trials in various clinical specialties and diagnostic studies the relevant obligations are very different, making an international study more difficult (Gluud et al., 2012). ECRIN, together with several organizations, advocate a risk-based regulatory and ethical oversight to ease the initiation of clinical trials not investigating new chemical entities. The OECD Global Science Forum works along the same principles and intends to globalize a concept essentially similar to that built in Europe (OECD Global Science Forum, 2011). Such global networking will be very helpful for involving many LMICs in large international non-industry sponsored clinical trials. In addition such regional network organizations might offer an excellent opportunity to perform studies of local medical relevance.

European Clinical Research Infrastructure Network (ECRIN) also champions a three tiered concept for training clinical investigators that was developed jointly with PhT. The first level contains all the basic ethical and scientific information needed for new clinical investigators as well as for the supporting hospital staff. The second level provides more complex information concerning trial management and regulatory requirements for experienced local chief investigators. Finally the third tier was conceived for coordinating investigators and deals with complex design of multicenter, multinational trials, together with the underlying international regulatory framework (Boeynaems et al., 2013). The Clinical Investigator Certificate (CLIC) educational concept can be very easily adjusted to the local conditions due to its stratified organization of knowledge.

In recent years clinical trials investigating targeted and advanced therapies considerably increased the scientific knowledge necessary for appropriate ethical review. The need for training of ethical committee members was repeatedly pointed out. In addition, Kerpel-Fronius mentioned that the increasing use of human biopsy materials in clinical trials requires appraising the impact of the planned research project on the human rights of the trial subjects. He recommended creating single united national ethical committees in LMICs instead of multiple committees working at the different universities. The major advantage of such centralized ethical evaluation of the clinical research projects is that the necessary expertise can be pulled together from the entire country. In addition a national ethical committee can better control that ethical principles are uniformly applied. Such a system works excellently in Hungary. However, it was pointed out by Massud that the experience with a central ethical committee providing only guidelines for the local active ethical committees is unsatisfactory in Brazil. It seems therefore to be important to elucidate further the possible advantages, the necessary statutes and organization for optimal scientific functioning of central ethical committees. Regional multinational ethical committees are not advocated due to the variations in the national legal framework, which makes joint ethical decisions practically impossible.

### Quality Assurance

The need for good a quality management system is of paramount importance for all levels of education. The quality principles worked out by the IMI Education and Training projects were presented by Dubois. They essentially state that the training should be offered on an equality basis, the courses should encourage a multidisciplinary approach and should be transparent regarding conflict of interest. The teaching staff should be well trained, and the methods and facilities should be adequate to support the acquisition of the aimed knowledge and competency (Klech et al., 2012).

Considering that e-learning is becoming more popular, additional quality standards were developed for this type of education. For broad application these programs should be platform independent, easy to use, reliable, securely defended by a firewall policy and, if required, could be applied for virtual classroom teaching. In addition they should fulfill the following e-quality standards:

• Blended levels of interactivity to be available. This can be either face-to-face combined with internet-based, or internet-based with integrated live activity.
• System in place for online practice exercises.
• Archiving policy to be defined to allow repeated viewings and/or spreading modules over time.
• System in place for obtaining feedback in conjunction with self-assessment questions (e.g., tutor availability to review questions after assessment is completed).
• Standards for tutorial activities (responsibilities, organization and implementation).

It was emphasized that adherence to these quality standards is essential at any place where an educational program is organized. Only qualitatively well-controlled education can provide internationally accepted certificates which are desired by the attendees of courses presented in LMICs. Finally it is evident that the joint efforts of the local and international teams are needed for the satisfactory compliance with these criteria.

## Discussion

A short workshop can only provide a sketch of the complex reality which must be filled in with the necessary details by subsequent activities of teams providing educational programs in LMICs. Nevertheless, the main outlines of the educational needs for developing and regulating medicines efficiently in emerging economies could be clearly formulated. It was unanimously accepted that parallel with the improving economy a rapidly increasing urbanized population emerges. They have a longer life expectancy and suffer increasingly from the same NCD present in developed countries. They need modern therapeutic interventions, including innovative and follow-on drugs introduced in richer countries. However, neither the health care and regulatory infrastructure, nor the number of well-educated personnel, is adequate to meet this enormous challenge. Therefore, the main focus of future education must be the rapid expansion of the number of various types of local experts trained in medicines development, regulations, and clinical research, respectively. These differently educated specialists should become able in a concerted effort to participate in the international and local development of new drugs, to guarantee their rapid regulatory acceptance and finally to apply them safely and efficiently at the bed side.

It was also pointed out by the participants that, to achieve these goals, training must be multidisciplinary for supporting equal development of these scientific disciplines. The education must be competence-oriented and fulfill the local needs. The medicine development, regulatory, and clinical research educational programs recently harmonized within the European IMI-PhT project can satisfy these expectations according to the experience obtained in differently developed regions of the world. The programs can be relatively easily transferred from Europe to any country since they cover all the important relevant topics, are organized in a separately presentable modular structure, and have defined module-specific learning outcomes leading to the desired competence. This conceptual organization is a great help in the proper alignment of the program with the local requirements since the different modules and topics can be easily rearranged and combined into the desired teaching program. Naturally, an additional adjustment to the educational background of the audience will be always needed. The importance of the availability of proper quality standards were emphasized by the participants since they are the guarantee for delivering internationally acceptable high level education in any country.

An important discussion point was to define the desired outcomes of the educational efforts. It was suggested that all the various programs should support the organization of scientifically well prepared program strategy teams, in which the partners are able to perform end-to-end product development. It was further emphasized that regional cooperation in all these scientific areas is of paramount importance. The experience of the WHO and several locally active organizations indicates that only a cooperative regional-multinational approach can efficiently bring together the number of differently qualified personnel necessary to handle rapidly complex issues. The participation of international experts in such regional working groups is not only helpful but is a very efficient approach to provide hand-on training to local colleagues. This suggestion is also true for the ethical review of increasingly complex clinical trials. International clinical research, regulatory and educational networks established in Europe and elsewhere might serve as useful examples to develop regional organizations also LMICs.

The final conclusion and message of the workshop is that the rapid economic and sociological changes which have occurred in the LMICs have transformed the health care and regulatory environment to an extent which makes the direct transfer of sophisticated knowledge acquired in the developed countries necessary and possible. Competence-oriented, modular educational programs for supporting these efforts are now available. They only need to be aligned with the local needs. To follow up this conclusion the PharmaTrain Federation and IFAPP formed a joint working group to translate this conclusion into practice. Efforts are underway to develop cooperation with local experts and institutions as well as with international organizations active in supporting health care in LMICs.

### Conflict of Interest Statement

The authors declare that the research was conducted in the absence of any commercial or financial relationships that could be construed as a potential conflict of interest. The Associate Editor, Jean-Marie. Boeynaems, declares that, despite being affiliated to the same institution as the author Dominique Dubois, the review process was handled objectively and no conflict of interest exists.
